# Odor Stimuli: Not Just Chemical Identity

**DOI:** 10.3389/fphys.2019.01428

**Published:** 2019-11-27

**Authors:** Mario Pannunzi, Thomas Nowotny

**Affiliations:** University of Sussex, Brighton, United Kingdom

**Keywords:** olfaction, odor stimuli, physics, chemistry, insect navigation, fluidodynamic

## Abstract

In most sensory modalities the underlying physical phenomena are well understood, and stimulus properties can be precisely controlled. In olfaction, the situation is different. The presence of specific chemical compounds in the air (or water) is the root cause for perceived odors, but it remains unknown what organizing principles, equivalent to wavelength for light, determine the dimensions of odor space. Equally important, but less in the spotlight, odor stimuli are also complex with respect to their physical properties, including concentration and time-varying spatio-temporal distribution. We still lack a complete understanding or control over these properties, in either experiments or theory. In this review, we will concentrate on two important aspects of the physical properties of odor stimuli beyond the chemical identity of the odorants: (1) The amplitude of odor stimuli and their temporal dynamics. (2) The spatio-temporal structure of odor plumes in a natural environment. Concerning these issues, we ask the following questions: (1) Given any particular experimental protocol for odor stimulation, do we have a realistic estimate of the odorant concentration in the air, and at the olfactory receptor neurons? Can we control, or at least know, the dynamics of odorant concentration at olfactory receptor neurons? (2) What do we know of the spatio-temporal structure of odor stimuli in a natural environment both from a theoretical and experimental perspective? And how does this change if we consider mixtures of odorants? For both topics, we will briefly summarize the underlying principles of physics and review the experimental and theoretical Neuroscience literature, focusing on the aspects that are relevant to animals’ physiology and behavior. We hope that by bringing the physical principles behind odor plume landscapes to the fore we can contribute to promoting a new generation of experiments and models.

## Introduction

### Olfaction, the Complex Sense

Animals use their olfactory system in almost every aspect of their life (e.g., to locate food, hosts, oviposition sites, and sexual mates, or to avoid predators). In order to properly investigate animals’ olfactory systems and their odor dependent behaviors we need to adequately define and characterize the sensory input received from the environment. For example, in the auditory system, a full understanding of the Doppler effect is needed to design experiments that meaningfully test echolocation by bats (Webster and Weissburg, 2001).

In color vision, the wavelength of light is the fundamental organizing property relating to color, and responses of cones in the retina, and subsequent color perception, can be well characterized as a function of the wavelength and intensity of light. In contrast, there is no single organizing principle for the space of all possible (volatile) compounds (Turin, 2002; Zhou et al., 2018) and the structure and dimensionality of odor space is an open problem^1^, to the extent that one may even wonder whether, in spite of the common element of involving the detection of chemicals, we can speak of a single sensory modality or not^2^. Furthermore, natural odors are often defined by numerous chemical compounds (odorants) (Raguso, 2008), present at a given concentration ratio, which compounds the difficulty of the problem. However, these aspects of odor space have been addressed before (for a review see, e.g., Sell, 2006; Auffarth, 2013) and we will not focus on them here.

Besides having the organizing principle of wavelength for light stimuli, we are also able to tightly control light stimuli in experiments with respect to wavelength, intensity and arrival time at sensory cells, allowing to build up deeper insights about visual perception (Hecht, 1942; Tinsley et al., 2016). Unfortunately, the same cannot be said about odor stimuli and in this review we will focus on two important aspects of the problem of *stimulus control*:

First, each odorant has different attributes in terms of its volatility (Cometto-Muñiz et al., 2003), how it distributes in the environment, adheres to surfaces, or dissolves in liquids or a carrier gas and, these attributes can change depending on environmental conditions such as temperature, pressure, humidity or even simply the characteristics of a container. When mixtures of odorants need to be considered, the complexity of the problem increases, both for the interaction between chemical components in the environment and for their interactions with receptors (see for example, Rospars et al., 2008; Wilson, 2013; Szyszka and Stierle, 2014). There are potentially serious consequences of not considering these properties. For instance, we might incorrectly assume that we can generate identical square inputs (or step stimuli) with different odorants, while in reality the concentration time course for each odorant is different because of its physical properties (Su et al., 2011; Martelli et al., 2013). Then, we likely would wrongly attribute observed properties of the response (e.g., latency) to the transduction process while it actually was a property of the odorant. These issues become even more problematic where neural responses are not simply proportional to the instantaneous concentration, but also strongly depend on its rate of change (Kim et al., 2011, 2015; Nagel and Wilson, 2011; Wilson, 2013).

Second, odorants are part of an environment that, most of the time, is turbulent and they form highly complex odor plumes (Murlis et al., 2000). Indeed, the spatio-temporal structure of odor plumes depends on both the physical properties of the airflow and on the odorants (Moore and Crimaldi, 2004). The properties of the flow determine the characteristics of the turbulence, while the properties of the odorants determine the interaction between diffusive and advective motion. The distribution of odorant concentration in space, with its valleys, crests, and plateaus, is commonly described as “odor-landscape” (Atema, 1996; Moore and Crimaldi, 2004; Celani et al., 2014). The matter, though incredible complex, does not lack beauty revealed through technologies that allow for ever better visualizations (see Van Dyke, 1982; Samimy et al., 2004).

A good knowledge of it is also indispensable to understand the plume exploration of insects (Murlis et al., 1992, 2000; Justus et al., 2002; Vickers, 2006). Only if we know what information (e.g., concentration, intermittency, variance of the concentration) is available to the insect at any given location in the plume, we might identify the potential mechanisms driving plume navigation. For instance, the details of surge and cast behaviors will depend on the statistics of odor filaments and suggested mechanisms for odor source separation (Baker et al., 1998; Sehdev and Szyszka, 2019) and can only be understood based on how correlations between odor plumes change depending on the separation between odor sources (Erskine, 2018).

The review was originally motivated by our work on formulating mathematical models of odorant receptors and the function of the early olfactory system in insects (Nowotny et al., 2013; Chan et al., 2018). Accordingly, we mainly focus on properties and situations that are relevant to insects and odor stimuli in air. We aim to raise awareness about the most urgent deficiencies in our knowledge and promote new thinking about how to design future models and experiments.

The organization of the review reflects the increasing difficulties of the discussed aspects of odor stimuli. In the first section, we will describe the difficulties in achieving a realistic estimate of odorant concentration and its time course “inside the lab.” In the second section, we will discuss the spatio-temporal structure of odorant plumes “outside the lab” up to the point of describing simple situations where mixtures of odorants are present. Each section will start with a summary of the related physical principles, followed by the review of the relevant Neuroscience literature. We will end with a brief general discussion.

## The Amplitude and Dynamics of Odor Stimuli in the Lab

“A philosopher once said, ‘It is necessary for the very existence of science that the same conditions always produce the same results’. Well, they do not.”

**R. P. Feynman**, *Lectures on Physics*, 1963

### The Physics of Dilution

R. Feynman was alluding to the lack of determinism of individual experiments in quantum physics, while reminding his audience of the need for reproducibility in science: Empirical experiments rest on the ability to characterize and generate controlled conditions in which to investigate a system of interest. In olfaction research, this translates to characterizing and generating defined odor stimuli. In essence, an olfactory stimulus is the presence of odorant molecules in the ambient medium (air, water) and is characterized by the identity and concentration of odor molecules at any given spatial location over time. The identity of odorants is easy to control in a laboratory setting (see, however, Andersson et al., 2012), but controlling the concentration is a much harder problem.

One common method for generating a controlled odor stimulus is the following: For each odorant, a preset amount of the pure odorant is placed into a container, usually a pipette. Odor stimuli are then delivered by injecting a preset fraction of the odor-laden air from the pipette into a stream of clean air for a preset stimulus duration. To use an odorant at a given concentration in air, experimenters dilute the odorant in a non-volatile solvent, i.e., paraffin oil (sometimes this solution is then applied to a piece of filter paper ∼1 cm^2^). The odorant will evaporate and fill the headspace of the pipette. While the variable of interest clearly is the concentration in air^3^, we only know the dilution of the original solution and it would therefore be useful to know the relationship between the dilution of the odorant in the solvent and the resulting odorant concentration in the air. When a compound (the solute) is dissolved in a liquid (solvent), a part of the compound evaporates. The amount of compound that evaporates depends on several factors, including the identity of the solute (odorant we are interested in) and the solvent, temperature, pressure, and the ratio between solvent and solute. Moreover, in the case of Pasteur pipettes and filter paper, even the interaction with the filter paper, the glass of the pipette and the air (in particular its humidity) affect the amount of evaporated compound.

**FIGURE 1:**
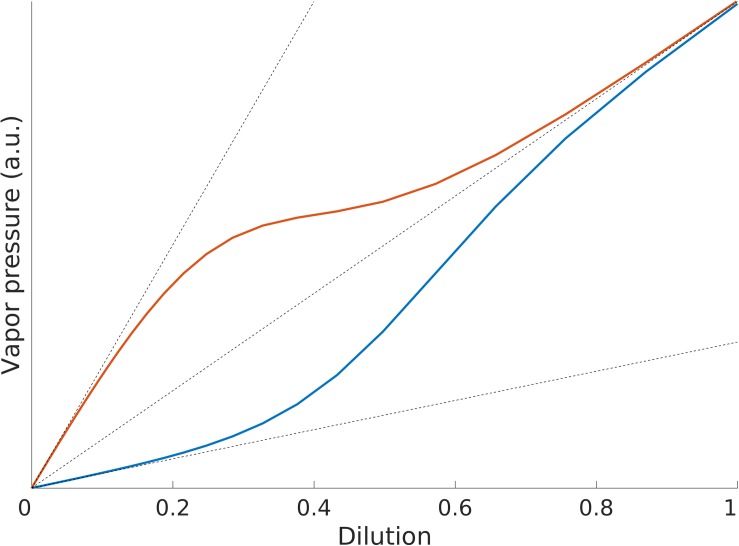
Illustration of Henry’s and Raoult’s laws. Both k_H_ > p^∗^ (red line) and k_H_ < p^∗^ (blue line) can be observed for different substances.

Without going into the details of the mechanisms governing gases dissolved in liquids here, we will focus only on the phenomenology of how the odorant concentration in the headspace of a solution relates to the dilution of the odorant in the solution. This relationship is generally described in terms of three regimes (see Figure 1): Henry’s regime (very low amounts of odorant), the intermediate regime and Raoult’s regime (very large amounts of odorant) (Gaskell, 2003). The odorant concentration/dilution relationship is monotonic in the three regimes, but it is linear only in Henry’s and Raoult’s regimes and the respective proportionality factors are different, Henry’s constant k_H_ for Henry’s regime and the vapor pressure of the pure odorant, p^∗^, for Raoult’s regime (see Box 1 and Figure 1).

**BOX 1. Volatility, dilution, and concentration.**

**Volatility**

Volatility, in chemistry, is the tendency of a substance to vaporize. The volatility of a substance depends on many factors, e.g., temperature, pressure, other substances within the same solution, etc. Volatility itself lacks unique quantitative descriptors, but “vapor pressure” and “normal boiling point” are commonly used as proxies for volatility:

If we put a liquid in a closed container, and wait long enough to obtain a (thermodynamic) equilibrium, then a part of the liquid will have evaporated. The resulting pressure in the closed container is the **vapor pressure** of this substance at the current temperature. Being another gas present, the substance partial pressure will coincide with its vapor pressure. If we add energy to the system – for example by increasing the temperature – the vapor pressure will increase (in a non-linear fashion following the Clausius-Clapeyron equation).

A volatile substance has a very high vapor pressure at “room temperature” (around 20°C). The temperature at which the vapor pressure of a substance is equal to the ambient atmospheric pressure is defined as its “**normal boiling point**.” Vapor pressure and “boiling point” are not independent, but roughly inversely related. Volatility correlates with a number of chemical properties, e.g., lipophilicity or hydrophobicity, i.e., a substance’s tendency to interact via van de Waals forces. Ultimately, at a microscopic level, the mechanisms that determine volatility are molecular mass and the quantum mechanical interactions between molecules.

**Henry’s law and Raoult’s law**

Henry’s law and Raoult’s law are empirical relationships between the dilution of a volatile in solution and the partial pressure *p* of the volatile in the head space above the solution. Using mole fractions, *x*, as the expression of dilution, Henry’s law can be written as: *p* = *x k_*H*_*, where *k*_*H*_ is Henry’s constant. This can be compared with Raoult’s law: *p* = *x p^∗^*, where *p*^∗^ is the vapor pressure of the pure volatile. More precisely, both laws are limit laws: Henry’s law is valid for extremely diluted solutions, while Raoult’s law applies at the opposite end of highly concentrated solutions. In mathematical terms:

$${\mathrm{Henry}}^{\prime }\,s\,\mathrm{law}:\lim_{x\to 0}p/x=k_{H}$$

$${\mathrm{Raoult}}^{\prime }\,s\,\mathrm{law}:\lim_{x\to 1}p/x=p^{*}$$

**FIGURE 2:**
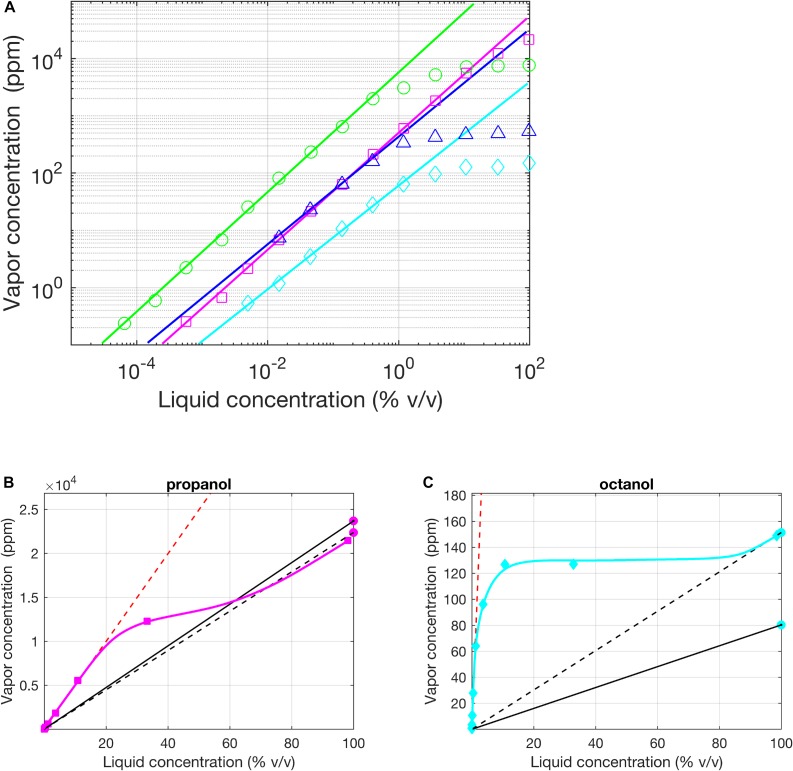
Regimes of Henry and Raoult, experimental evidence. **(A)** Vapor (p.p.m. by volume) vs. liquid (% v/v) concentration for homologous alcohols in logarithmic coordinates (1-Butanol – red circles, 1-Propanol in H_2_O – green squares, 1-Hexanol – blue triangles, 1-Octanol – cyan diamonds). Except for Propanol, the solvent was mineral oil. The solid lines indicate a fit of Henry’s law to the lowest concentration values (all slope 1 in the log-log plot, indicating linear fits). **(B,C)** Same as **(A)** in a plot with linear axes for propanol in H_2_O and 1-Octanol in oil. The two black lines (dashed and solid) are the fits for Raoult’s law using the average values of vapor pressure reported by the UCSD (California) lab and UCL (London) lab, respectively. Red dashed line is the linear fit of the Henry’s Law done with the data of Cometto-Muñiz et al. (2003) (approximating β equal 1). Modified from Cometto-Muñiz et al. (2003).

The vapor pressure p^∗^ is a single value for each substance and is reported widely in chemistry databases^4^. However, the values of the vapor pressure reported by different laboratories can actually be quite disparate (see Figure 2). Cometto-Muñiz et al. (2003) compared the reported values of vapor pressure for 36 chemicals by a group of authors working at the University of California and a group of authors working at the University College of London; for 16 chemicals, differences between values were larger than 25%. In the same study, the authors pointed out the limit case of the *octanal* vapor pressure whose reported value in text-books spans from 0.0053 mmHg to 2.14 mmHg (Cometto-Muñiz et al., 2003).

Henry’s constant depends on both the solvent and the solute and it is therefore generally more difficult to find. A valuable exception is the study of Cometto-Muñiz et al. (2003) in which the authors measured and reported k_H_ for several odors dissolved in mineral oil, the most commonly used solvent in olfaction research. Moreover, Cometto-Muñiz et al. also reported the extent of Henry’s regime for each odorant (see some examples in Figure 2).

When delivering odors using liquid solutions it is necessary to establish whether Raoult’s or Henry’s law apply or whether the dilution falls into the transition region. Frequently, all three situations apply to different parts of the same experiment if dilutions are varied (see Table 1 in Cometto-Muñiz et al., 2003). The difference between k_H_ and p^∗^ and, consequently, of the odorant concentrations estimated based on assuming either regime, can be very high (see Table 1 for a few relevant examples). Therefore, identifying the correct regime is very important.

**TABLE 1:** Example values for vapor pressure and Henry’s constant in mineral oil extracted from Cometto-Muñiz et al. (2003).

	**p^∗^[mm Hg]**	**k_H_**
Ethyl acetate	94	87434
Methyl acetate	235	148568
Geraniol	0.03	1
1-hexanol	0.92	429
1-octanol	0.2	60
Butyric acid	0.43	189
Pentanoic acid	0.2	50
Hexanoic acid	0.043	6.51

The investigation of the role of odorants in mixtures is a typical example where careful consideration of the relationship concentration/dilution is crucial. In this case, one would want to create mixtures with well-controlled concentration ratios, e.g., a mixture that contains the exact same number of molecules of each type. To do this, one needs to establish which regimes apply (it could well be different regimes for each of the odorants involved) and what the values of k_H_ or p^∗^ are for each of the involved odorants. We will revisit the question of controlling concentration by dilution later on when discussing concrete examples from olfaction research in insects.

### The Physics of Adsorption and Desorption of Gases on Solids

When passing through an olfactory stimulation device, volatiles will interact with the surface of the device’s air ducts. Some of the odorant (adsorbate) will adhere to the surface (adsorbent) and then detach from it again under physical (i.e., van der Waals force) or chemical forces (Rabe et al., 2011). This is the phenomenon of adsorption/desorption of a gas to a solid (Shirtcliffe, 2008; Foo and Hameed, 2010). Many different models have been formulated to describe the mechanism and the dynamics of adsorption; their detailed description is beyond the scope of this review, but, thanks to a renewed interest in adsorption for environmental reasons, it can easily be found elsewhere (e.g., Foo and Hameed, 2010). In essence, the models pursue the description, at thermodynamic equilibrium, of the amount of adsorbate as a function of the relevant parameters of the system, including the partial pressure of the adsorbate, the temperature and surface area, and, of course, the chemical properties of adsorbent and adsorbate. The most common models of adsorption adopt the hypothesis of constant temperature, so-called isotherm adsorption models, and interpret the process of adsorption as minimizing the surface free energy of the combined solid/gas system. The simplest of these models (valid at very low partial pressure of the adsorbate) predicts that the fraction of adsorbed adsorbate X is a linear function of its partial pressure p, X = H_k_ p. The proportionality factor is called Henry’s adsorption constant H_k_, named for the similarity to Henry’s law discussed above. The Langmuir model (Langmuir, 1932), was the first attempt of a semi-analytical model, and allowed to derive a rational function *X* = *H*_*k*_*p*/(1 + *H*_*k*_*p*). For low partial pressure this law is reduced to the linear model. Some of the assumptions of this model are perfectly realized in real-life scenarios, except for the simplifying assumption used that adsorbates would form only a single layer on the surface of the solid. More recent models attempt to deal with this complexity, but they do not yet succeed in providing a complete description of the phenomenon (Foo and Hameed, 2010). For the purposes of this review, all models indicate a monotonic relationship between the partial pressure of the adsorbate and the amount of adsorbate on the surface that additionally depends on the chemical properties of the adsorbate and adsorbent. In olfaction experiments, when volatiles pass through an olfactory stimulation device, the models hence predict a dependence of the concentration flow on an odorant’s partial pressure and its chemical identity, the former in turn being a function of odorant dilution with the two linear regimes (Henry’s and Raoult’s regime) as discussed above^5^.

### Application to Insect Olfaction Research

We will now proceed to review the relevance of the physics of odor delivery for designing and interpreting experiments. We will illustrate the key issues on a few typical research questions pertinent to olfaction research, for example the *relevance of different odorants* for any given insect.

#### Concentration of Odor Stimuli

To assess the *relevance of different odorants* for insects it is sensible to compare the physiological and behavioral responses to odorants of interest. To enable meaningful comparisons, the odor stimuli need to be of “comparable strength.” However, “comparable strength” can have different meanings depending on the objective of the study. For example, if one is interested in the ecological relevance of some specific compounds for an insect, one should determine the typical concentration of the compounds in natural settings and analyze the insects’ behavior with those concentration values. On the other hand, if one is interested in the general response of receptor neurons to different chemical compounds, then the choice for a fair comparison is typically to generate stimuli that deliver the same number of molecules within the same timespan to the antenna of the insect. However, as we discussed above, this quantity is not under the experimenter’s direct control. In order to achieve the right concentration at the antenna, experimenters need to reverse-engineer the correct dilution using the limit laws discussed above (see section The Physics of Dilution), and, potentially, considering differences in adsorption along the odorants’ path through the olfactometer (see section The Physics of Adsorption and Desorption of Gases on Solids). While this can be an arduous process, in particular if essential information about Henry’s constant and the properties of adsorption for any given stimulation device are missing, we believe that it is important, because the observed relevance of an odorant will depend substantially on getting the stimulus right.

Resource constraints often mean that only a single concentration per odorant can be sampled, which makes correct stimulus design even more important. The incredible effort of DoOR, for example, where the responses of *Drosophila* olfactory receptor neurons (ORNs) to a large number of odorants are collected and normalized in order to have a “single consensus response matrix,” has so far only been possible for a single concentration for each odorant (Galizia et al., 2010; Münch and Galizia, 2016). Similarly, the analysis of more than 100 odorants on 31 ORNs of Hallem and Carlson (2006) was only possible for one concentration of each odorant. Given these constraints, it would be valuable if a common process could be used to determine the correct dilutions for odorant stimulation that maximize the accuracy of comparing results. Rescaling after the fact (Galizia et al., 2010; Münch and Galizia, 2016) is a good first step but the many non-linearities in both, the physics of dilution/concentration and the early olfactory system may limit the validity of this approach.

Ideally, one would want to map the entire response profile of the insect olfactory system across different odors and different concentrations, as, for instance, pioneered in the work of Sachse et al. (1999), in which the authors performed the first systematic calcium imaging in the antennal lobe (the second phase of olfactory integration in insects) of bees with stimuli from the alcohol series (pentanol, hexanol, and so on to decanol). This allowed the systematic comparison of responses along the dimension of carbon chain length and across three different dilutions (1–10–100%). Vapor pressure decreases monotonically with carbon chain length, so that proportionately different odor concentrations would have reached the antenna of the bees for the 100% non-diluted odorants, for which Raoult’s regime applies. To account for this, we can try to compensate by dividing observed responses by the vapor pressure (assuming sufficiently linear properties of the olfactory response). For higher dilutions of 10 and 1%, however, Raoult’s regime is unlikely to apply (see, e.g., 1-butanol, 1-hexanol, and 1-octanol in Figure 2) and neither is Henry’s regime, which starts somewhere beyond 1% dilution. In essence, there is no straightforward way to compensate for the unknown non-linear relationship between dilution and concentration and the interpretation of results is very difficult.

Another pertinent example where the relative concentrations of odorants are very important is the *investigation of odor mixtures*, both in the pheromone sub-system and the general olfactory system. In the pheromone sub-system, it is well-documented that females of related, but sexually incompatible, moth species may use the same substances in their pheromone blends but in different concentration ratios (see, e.g., Christensen et al., 1989; Baker, 2008) and references therein). In order to find a compatible female, male moths need to recognize the blend when encountered in the air during upwind flight (Zavada et al., 2011). Arguably, the quantity relevant to this situation is the concentration ratio as generated in the glands of the female moth, which presumably is conserved in the environment. When generating diluted versions of the blend in the lab, dilutions of the individual pheromone components would need to be adjusted so that the resulting blend in the headspace has the correct concentration ratio: Different components need to be diluted differently if their regimes and proportionality constants (k_H_ and p^∗^) differ (see Table 1).

These considerations also become important when considering overshadowing (e.g., Schubert et al., 2015). Overshadowing is a phenomenon where bees conditioned by pairing a mixture AB with sugar water later respond more to odor A than to odor B when the odors are presented alone. Odor A appears to overshadow odor B in the perception of the mixture. To make a fair comparison between the two odorants in the mixture, we should use dilutions for odorants A and B that are inversely proportional to their vapor pressures, if Raoult’s regime applies, e.g., a dilution ratio of octanal and 2-non-anone of 0.52. However, Raoult’s regime is not very wide (see Figure 2) so that when using dilutions of 10% or more, octanal and 2-nonanone dissolved in mineral oil are already in Henry’s regime (Cometto-Muñiz et al., 2003) and their dilution ratio should be 0.62, a small but potentially significant difference. For other odorants and solvents, the difference could be much larger, depending on the values of k_H_ and p^∗^. Making the right adjustments is, however, only possible when these values are known, which is often not the case.

A possible approach to generate suitable odorant concentrations in air, albeit tedious and laborious, is to choose dilutions of odorants for experimental stimuli using the following procedure: (1) Measure the odorant concentration in the air at the antenna with a high resolution detector (see below) for different values of dilution, (2) Determine which regime the odorant solution is in for the dilution values that are relevant to the problem at hand, (3) If one of the linear regimes applies, extract the value of the relevant proportionality factor (the vapor pressure or Henry’s constant), and (4) Use the odorants at a dilution that is inversely proportional to this relevant factor. Unfortunately, depending on the experimental conditions, this procedure may or may not be sufficient. One of the complications is the detector. Nowadays, the fastest detectors are those using photoionization technology, PIDs (photoionization detectors). In these detectors, a UV light source ionizes airborne molecules and the charge produced by ions is measured by the instrument. The PID measures concentrations down to low concentrations (∼few parts per billion) and a relatively high sampling rate of hundreds of Hertz (for an extensive analysis of detectors see e.g., Riffell et al., 2008). However, PIDs, like other analytical chemistry tools, e.g., gas chromatographs, do not report absolute values of concentration, but have to be calibrated to obtain this information. For PID calibration, some studies have used the *known* concentration of an odorant as a reference (e.g., Kim et al., 2011, 2015) which shifts back the problem to an initial calibration of this concentration. Alternatively, PIDs were calibrated assuming to know the concentration based on a theoretical approach, using Raoult’s and Henry’s law for odorants diluted in a solvent (Olsson et al., 2011); or for pure odorants, simply Raoult’s law (e.g., van Breugel and Dickinson, 2014); of course, this approach can be affected by the problems related to the inconsistency of vapor pressure (see above and Figure 2). In a recent attempt (Gorur-Shandilya et al., 2019) proposed to calibrate PIDs based on the measurement of known masses of chemicals (similar to gas chromatograph calibration).

Until now, we have neglected another very important variable: time. We have analyzed the system in terms of a thermodynamic equilibrium, neglecting the dynamics of the processes involved. This pertains to the thermodynamic processes of evaporation as well as the dynamics of removing odor laden air from the stimulation device in order to expose the animals to it. We will discuss the latter aspect in the next section, but conclude this one with an issue related to the processes of evaporation. A clear demonstration of the risks of repeatedly using a finite amount of odorant that depletes with time is shown in Andersson et al. (2012). The authors showed that the depletion of commonly used odorants depends strongly on the volatility of the odorants. The depletion experiment they used was designed to replicate the typical day (8 h) of neurophysiological experiments in olfaction research: Each odorant was emitted every 10 min for 50 times (or until its concentration was below response threshold). They found that each individual compound has a characteristic time-scale of odorant depletion and that for many of the tested compounds the odorant concentration depletes more rapidly than naively expected, e.g., to almost zero in only two puffs. This issue can, for example, be relevant when characterizing response specificity and sensitivity of ORNs. When correcting for depletion effects (Andersson et al., 2012) found, contrary to earlier reports of comparable responses to all three odorants (Stensmyr et al., 2003; Hallem and Carlson, 2006; Pelz et al., 2006), that the ab3A receptor in *Drosophila* is highly specific to ethyl hexanoate, and orders of magnitude less to methyl hexanoate and ethyl butyrate. To avoid the issue of depletion few adjustments should be and nowadays are applied: (1) Taking the odorant-saturated headspace of a sufficiently large reservoir of pure odorant, (2) Using a much larger headspace volume than the stimulus-volume in order to avoid measurable dilution of the odorant with air when replacing the removed odorant volume, and (3) Using a device (e.g., Mass Flow Controllers) to regulate the air flow removing the odor from the headspace in order to regulate the odorant concentration. The superior stability of repeated odor stimuli obtained with these adjustments can be seen in Gorur-Shandilya et al. (2019).

#### Temporal Structure of Stimuli in the Lab

Temporal patterns of neural activity in the antennal lobe are hypothesized to play an important role in olfactory coding (e.g., Laurent and Davidowitz, 1994; Brown et al., 2005; Mazor and Laurent, 2005; Wilson et al., 2017). These temporal patterns originate from at least two separate sources. They reflect the temporal pattern of the odor stimuli arriving at the antenna, and they emerge from the internal network dynamics in the recurrent antennal lobe network. To achieve an accurate description of the temporal aspects of neural responses, it is therefore essential that we have a precise control over, or at least a measurement of, the temporal properties of olfactory stimuli.

One of the most common stimuli in psychophysics is the step function: A stimulus, for example a flash of light or a sound is emitted for a duration of interest, with a constant amplitude. The advantages of using such simple stimuli in a reductionist approach are clear, in spite of their hidden complexity: the instantaneous step from 0 to x implies the use of all frequencies. Visual and auditory step stimuli have been studied for a long time and we know their properties very well, but what happens when considering rectangular steps for odor stimuli?

Many studies have analyzed insects’ neural responses to chemical compounds, using an approximation of step stimuli in conjunction with electrophysiological recordings or calcium imaging (e.g., de Bruyne et al., 1999; Hallem and Carlson, 2006; Galizia et al., 2010; Münch and Galizia, 2016). In these experiments, odor stimulation pipettes are prepared with a diluted odorant. A stimulus is then generated by passing an air puff through the pipette to transport the volatile molecules to the olfactory receptors. Once the valve controlling the odorant pathway is open, the volatiles start to flow and eventually reach the olfactory sensilla on the antennae. At least two processes separate the odorant in the pipette from arriving at the receptors: passing through the stimulation device and bridging the gap from the exit of the stimulation device to the antennae, through the open air. These processes cannot be characterized as simple fixed-time delays for odorant arrival; their durations depend on many factors, for example the chemical structure of the solvent, the dilution, the storage conditions, the puff interval and puff number (Andersson et al., 2012), the airflow, the tube diameter, the distance of the insect from the tube exit, the distance from the pipette to the exit of the stimulation device, and the lateral distance from tube axis may all affect the temporal integrity of the stimulus (Vetter et al., 2006).

Evidence for the relevance of the odorant pathway through the stimulation device was presented in Nagel and Wilson (2011) and carefully analyzed in Martelli et al. (2013) and Su et al. (2011). These two studies demonstrated that the resulting stimulus dynamics can depend on odorant identity, but typically not on the odorant concentration. Furthermore, they demonstrated how the stimulus dynamics for almost 30 odorants (chosen for their ecological relevance for flies; Hallem and Carlson, 2006) can be described with an onset and an offset timescale and that these timescales are correlated with the vapor pressure of the odorants (Martelli et al., 2013). It is striking that even for this comparatively small sample of chemical compounds the variability of timescales is enormous, spanning 2 orders of magnitude from 30 ms to 1 s. This highlights the fallacy of the abstraction of a step stimulus for odor stimuli. An extensive analysis of the mechanisms behind these processes is still missing, but the large and strongly disparate deviations from an instantaneous step are likely due to the different adsorption/desorption dynamics inside the stimulation device experienced by different compounds and at different partial pressures (as previously noted by Martelli et al., 2013). However, it is important to note that it is unlikely that the relevant quantity is the vapor pressure. If adsorption/desorption is to blame, the relevant property is probably Henry’s adsorption constant H_k_ (see section The Physics of Adsorption and Desorption of Gases on Solids), which offers a potential explanation why time-scales at times appear to scale non-linearly with the vapor pressure (Martelli et al., 2013).

It is worth noting at this point that the dynamical nature of stimulus arrival at the antennae is not only highly relevant when analyzing neuronal and behavioral response times. It also can change the response amplitude because the responses of ORNs, and subsequently of the projection and local neurons in the antennal lobe, are not simply proportional to the total amount of volatiles bound, but also strongly depend on the rate of change of bound volatiles (Kim et al., 2011, 2015; Nagel and Wilson, 2011; Wilson, 2013). Therefore, not only is it problematic that we lack clear information on the concentration of the stimuli, but it is equally, if not more, damaging that we often do not know the rate of rise and decay. A direct comparison between neurophysiological or behavioral responses for stimuli with different rise and decay time constants, without proper rescaling, risks misinterpretation of the data and proper rescaling can only be achieved when measuring the vapor concentration time series at the antenna (e.g., Kim et al., 2011; Nagel and Wilson, 2011; Martelli et al., 2013). Further investigation of these issues may well impact on our interpretation of the existing data as, for instance, collected in DoOR (Galizia et al., 2010; Münch and Galizia, 2016) or as reported in experiments looking at the roles of odorants in mixtures (Su et al., 2011; Schubert et al., 2015; Chan et al., 2018).

The ultimate goal of olfaction research in neuroethology is to understand animals’ senses as they are relevant to their behavior in a natural environment. In order to do so, researchers attempt to recreate realistic stimuli in the lab under controlled conditions. But what is a realistic “spatio-temporal structure” of an odor plume? In the next section, we will review results of experiments and theory on the distribution of odorants in natural environments outside the lab.

## The Spatio-Temporal Structure of Odor Stimuli in a Natural Environment

“There is a physical problem that is common to many fields, that is very old, and that has not been solved. It is not the problem of finding new fundamental particles, but something left over from a long time ago – over a hundred years. Nobody in physics has really been able to analyze it mathematically satisfactorily in spite of its importance to the sister sciences. It is the analysis of circulating or turbulent fluids.”

**Richard P. Feynman**, *Lectures on Physics*, 1963

### The Physics of Odor Plumes

As described in the quote of R. Feynman, the physics of plumes is extremely complex and, even though incredible advances have been made over the past 50 years, we still cannot claim to have a complete description of the phenomenon. Consequently, we will not be able to treat this problem in its full difficulty but we will try to summarize the aspects of plume structure that are most relevant for olfaction.

Generally, the physics of fluids is described by non-linear partial differential equations, the Navier-Stokes equations. In the context of odor plumes, scientists commonly assume incompressible fluids, and so can use the simplified incompressible Navier-Stokes equations. However, even the simplified equations are analytically intractable for most real life problems (Shraiman and Siggia, 2000; Falkovich et al., 2001) and research relies on numerical simulations and empirical measurements in the field.

We will refer to theoretical works to describe the most relevant physical properties that can affect the odor landscape, but discussing them would go beyond the aim of this review (for excellent reviews of the theoretical literature (see e.g., Shraiman and Siggia, 2000; Falkovich et al., 2001). The first and most important distinction in the dynamics of flows is between laminar and turbulent flows. Turbulent flows are characterized by chaotic fluctuations of flow speed and pressure. Eddies and vortices are the typical pictorial representations of turbulent flows; laminar flows, on the contrary, reflect reversible behavior stemming from simpler parallel movements. The transition from turbulent to laminar regime is determined by the balance between viscous and inertial forces. High viscosity drives the flow toward a laminar condition and high inertial forces toward turbulence. The Reynolds number (Re) is essentially the ratio between these two kinds of forces, and hence describes this balance, even though without a clear cutoff value for the transition between turbulent and laminar regimes (see Box 2). The factors determining Re are the viscosity of the fluid (higher viscosity, less turbulence), the density of the fluid (higher density, higher turbulence), and the speed of the flow (higher speed, higher turbulence). The last discriminative factor is the *characteristic spatial scale* of the system: If we want to determine the turbulence of a fluid flow in a pipe, the characteristic length is the pipe diameter; while if we are interested in the air flow around an insect, the characteristic length can be estimated as the diameter of the insect. While Re is commonly calculated on average values of flow speed, we have to keep in mind that the flow speed can vary throughout the analyzed system. In particular, the speed of fluid layers close to a solid surface depends on the height: It is approximately zero in the layer in contact with the solid surface – as adhesion induces a no-slip condition – and then increases logarithmically with height until reaching the average wind speed. The region close to the surface is called a boundary layer and when the surface is the Earth’s ground, it is the atmospheric boundary layer (see Box 2).

So far we have essentially described the flow of a single fluid. In the context of olfaction we need to analyze a more complex situation of a fluid (the odor) immersed in another fluid (the background atmosphere/ambient air). The spatio-temporal distribution of “odor fluid flow” is determined by the fluid dynamics of the ambient atmosphere (with Navier-Stoke equation see Box 2) and the motion of the odorant within it (Shraiman and Siggia, 2000; Falkovich et al., 2001). The equation that governs the dynamics of the odorant concentration inside the air (or water) flow is the advection-diffusion equation. The name is self-descriptive and refers to the two physical processes underlying it: advection – bulk motion – and diffusion – Brownian motion. The balance between these two processes is described by the Péclet number (Pe) (see Box 2), which is the ratio between the rate of advection and the rate of diffusion.

In summary, odor sources, including organic (animals, plants, or their decay products), geological (Volcanoes) and man-made sources, emit odorants in the air where they travel driven by advection, and molecular diffusion on the background of the airflow, which can be turbulent or laminar in nature. The interaction of these transport processes generates the odor-landscape, that is the distribution of the odorant concentration in the air (Atema, 1996; Moore and Crimaldi, 2004; Celani et al., 2014).

#### Main Features of Odor Landscapes

**FIGURE 3:**
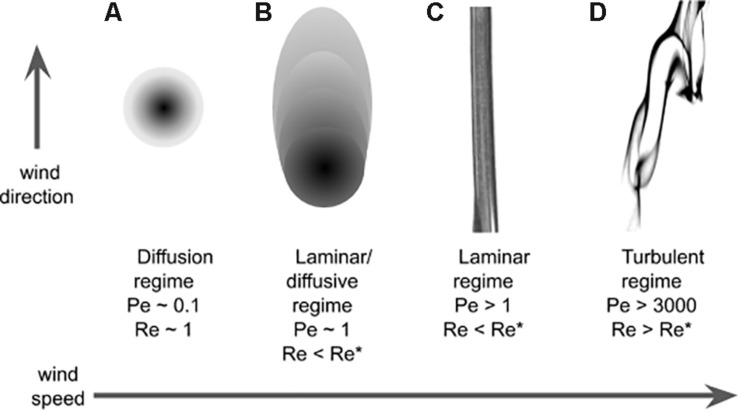
Plume structure. Regimes that are qualitatively different for increasing value of wind speed. The regimes are identified for different values of Péclet and Reynolds numbers: **(A)** Diffusive regime, **(B)** laminar/diffusive regime, **(C)** laminar regime, and **(D)** turbulent regime (see text). In this pictorial representation the Schmidt number (Pe/Re) is around 0.1.

The main feature that characterize an odor landscape in diffusive and laminar conditions (Figures 3A,B) is the (smooth) odorant concentration. It is therefore not surprising that concentration gradients are used for chemotaxis by very small insects at low Reynolds and low Peclét numbers. In the more complex turbulent regimes (Figures 3C,D), the most salient feature of the odor landscape is probably its patchiness: with the exception of habitats with low Reynolds number and where diffusion can be stronger than advection, odor concentration lacks a continuous, let alone smooth, spatio-temporal distribution (see Figure 3). It therefore proved useful to describe it in terms of *filaments* (or odor-strands), i.e., pockets of non-zero concentration of odorant, or in the temporal domain in terms of *whiffs* – time intervals with non-zero odor concentration – and the complementary concept of *blanks* – time intervals with zero odor concentration. In addition the odor landscape is typically described in terms of variables such as the average concentration $\bar{C}$ and the (temporal) fluctuations of the concentration^6^ $\sigma_{C}/\bar{C}$, where σ*C* is the standard deviation (over time) of the concentration. These and other variables were analyzed with respect to whiffs and blanks, defining “conditional measures” as the mentioned measures restricted to within whiffs. For example, the conditional average concentration is the average concentration during whiffs. The discontinuous nature of whiffs and blanks is typically called *intermittency*. There are different definitions in use. Here we use the definition of intermittency (or intermittency factor) *x* as the fraction of whiff time, i.e., high intermittency means many whiffs or long whiffs.

Because the viscosity and density of air does not change dramatically, the main factors related to the nature of odor plumes in (turbulent) natural air flows are the average wind speed and its fluctuations, the physical space, e.g., open field vs. forest, and the height above ground, both of the odor source and of the animal smelling it. An additional factor is the time of day, which determines the buoyancy in the atmospheric boundary layer and hence the balance between turbulence caused by buoyancy vs. turbulence due to wind shear.

With respect to the advection/diffusion balance, the most important factors are the nature of the odorant, in terms of its diffusivity (reflected in the Péclet number, see Box 2), whether it is a simple compound or a complex mixture, or something in between, such as pheromones.

**BOX 2.Fluid dynamics.**

Fluid dynamics distinguishes two regimes: Turbulent flow, when pressure and flow velocity behave chaotically and laminar flow, when the fluid flows in parallel surfaces. Laminar flows are characterized by high viscosity and/or low kinetic energy. Transitions from laminar to turbulent flow can, for instance, be observed in the smoke of a flame, at few centimeters distance from the flame.

A complete analytical description of turbulence is still beyond our grasp and is included on the list of unsolved problems in physics. Physicists and engineers analyze most real-life turbulent flows through numerical analysis with computational fluid dynamics models.

The **Navier-Stokes equation** describes the motion of fluids under diffusing viscous forces.

The **Reynolds number (Re)** is defined as the ratio between inertial and viscous forces experienced by a solid body moving in a fluid (e.g., a fly in air), or, equivalently, as caused by a fluid flowing around a stationary solid body (e.g., air around a tree trunk). *Re* = *U*/*ν*/*L*, where *U* is the advective speed, ν is the kinematic viscosity and *L* is the characteristic length.

The Reynolds number is used as a rough guide for the expected nature of the flow. A flow is laminar for low values of Re, and it is turbulent for high Re. For the flow in a pipe, low Re values are commonly below 10^3^, but there is no precise number that marks the transition. **During turbulent flow, the fluid’s fluctuations in speed and direction, are high and around the same order of magnitude of the average wind speed**. It can be instructive to see the Re value for a typical situation, e.g., a windtunnel with a diameter of 40 cm and wind speed around 0.5 m/s. The other relevant quantities are the dynamic viscosity of air (∼18.5 μPa⋅s) and the density of air at 20°C (∼1.2 Kg/m^3^). In this situation Re ∼ 10,000, the threshold value Re^∗^ is around 3000. **Advection-diffusion equation** describes how a physical scalar quantity, such as mass or heat, varies in time in a fluid flow. For example, in our case, odorant concentration, c, varies for the variation of the flux **j** of the odorant and depending to an external source (or sink) *R*:

$$\frac{\partial c}{\partial t}=-\nabla \cdot j+R$$

The flux results from the sum of a diffusive and an advective term. The “diffusive flux” due to random Brownian motion of molecules is typically approximated to the gradient of the local concentration: *j*_*diff*_ = −*D*∇⁡*c* where *D* is the molecular diffusivity that depends on several parameters, among them the temperature, the pressure, the molecular mass of both air and odorant diffused. The advective flux is due to a net bulk motion driven by the wind with speed ***v***: *j*_*adv*_ = *vc*

**Péclet number** (Pe) indicates the separation between flows that are dominantly diffusive from advective ones for a scalar variable governed by the convection-diffusion equation.

**Re** = *U/(D/L)*, where *U* is the advective speed, *D/L* is the diffusion rate, *D* is the molecular diffusivity and *L* is the characteristic length.

For Pe smaller than one, diffusion dominates otherwise advection dominates. For example, for pheromones, that are small volatile compounds, whose the diffusion coefficients are of the order of 10^–6^m^2^/s their Peclét number exceed unity by several orders of magnitude (Cardé and Willis, 2008) in typical conditions – wind speed around 1 m/s and **L** of ten or more meters.

The **Schmidt number** is the ratio between Péclet number and Reynolds number, that is the ratio between viscosity and the product of the density of the fluid and the diffusivity of the odorant in the air.

**Batchelor scale** indicates the smallest length scale at which fluctuations in scalar concentration take place before molecular diffusion dominates the dynamics $\lambda_{B}{=}^{4}\sqrt{D^{2}\,\nu /\varepsilon }$, where *D* is the molecular diffusivity, ν is the kinematic viscosity, and ε is the mean viscous dissipation rate.

The layer of fluid close to the surface, where viscosity is strong, is called **boundary layer**. When the surface is the Earth’s ground, the air layer is the **atmospheric boundary layer**. The flow speed in this layer depends on the height: It is approximately zero at few millimeters from the ground for the viscosity determine a **no-slip condition** there and then it increases logarithmically with the height until reaching the average wind speed.

For both, the nature of the flow and of the odorant transport in the flow, scale and distance matter. The observed characteristics of the plume change with the distance from the source, either down-wind, or cross-wind, and with the size of the source and receiver.

#### Results on Plume Structure

“Any experiment is reproducible until another laboratory tries to repeat it.”

**Kohn’s Second Law**

In the following sections we will review work on aspects of plume structure, focusing on those aspects that are most relevant to animals: average concentration, concentration fluctuation, intermittency and whiff and blank durations. Ideally, we want to give enough information for an experimenter to reproduce, using an odorant stimulator, what could pass as natural stimuli. When investigating aspects of plume structure in the field, scientists typically place an odor source at a defined height and measure the odorant concentration time series at defined locations downwind/crosswind, using a detection device, for example a PID.

##### Average concentration

Mylne and Mason (1991) used propylene as a tracer gas to measure the concentration averaged over time, in neutral buoyancy conditions, at large distances of tens to hundreds of meters, with source (1 cm diameter) and detector 2 m above the ground and in an open field, flat and smooth for several km in all directions (Mylne and Mason, 1991). They found that, as one might expect, the average concentration decreases with the downwind distance from the source. This was also seen in other studies (e.g., Voskamp et al., 1998; Murlis et al., 2000) and for smaller distances from the source (from a few meters to 30 m), and in wind tunnels (Fackrell and Robins, 1982a, b; Justus et al., 2002; Vergara et al., 2013). Commonly used Gaussian plume models predict that the average concentration on the midline of the plume decays with a power law, with data fits indicating powers between -1.5 and -2 (Cramer et al., 1958; Fares et al., 1980).

**FIGURE 4:**
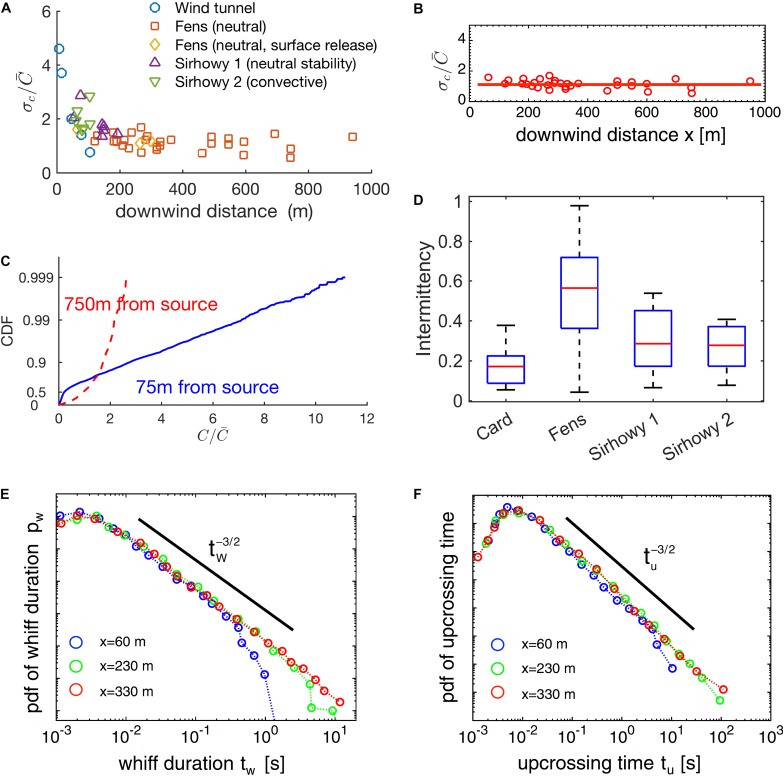
Odor-landscape in the atmospheric boundary layer described by the statistics of its principal properties. **(A)** Fluctuation of odorant concentration $\sigma_{C}/\bar{C}$ for several distances from the source and for several conditions (see Mylne and Mason, 1991). **(B)** Subset (“Fens” dataset) of data reported in **(A)**. **(C)** Cumulative distribution function of the concentration for two samples at 75 and 750 m distance from the source. **(D)** Probability distribution of intermittency for several locations. The theory can partially model the experimental results reported by Yee et al. (1995). **(E,F)** Probability distribution functions for whiff durations and upcrossing durations (data from Mylne and Mason, 1991; Yee et al., 1995). **(B,C,F**) Reproduced from Celani et al. (2014), **(A,C,D)** reproduced from Mylne and Mason (1991).

The shape of the *probability distribution* of concentration on the centerline has been argued to (Hanna, 1984; Lewellen and Sykes, 1986; Mylne and Mason, 1991) vary as a function of downwind distance as well (75–75 0 m, see Figures 11 and 13 in Mylne and Mason, 1991; Figure 4C).

However, *for very small distances* (a few meters from the source), *only* the probability distribution of concentration on the centerline of a turbulent jet appears to depend on the downwind distance (and other experimental parameters), but the average concentration *does not* (Duplat et al., 2010).

##### Concentration fluctuation and intermittency

The fluctuations of concentration $\sigma_{C}/\bar{C}$ (*both conditional and not*) decrease with downwind distance, steeper close to the source and more gradually at large distances. This result was consistently demonstrated, albeit with large variability of individual measurements, in a large number of experiments (e.g., Mylne and Mason, 1991; Mylne, 1992; Yee et al., 1993; Mylne et al., 1996) for long distances (>20 m), in open field conditions, during near-neutral and stable buoyancy conditions. It was also observed for shorter distances (5–20 m) with similar meteorological conditions (Davies et al., 2000) and in small wind-tunnels (3 m) (Fackrell and Robins, 1982b; Vergara et al., 2013).

In crosswind direction, at a given downwind distance, fluctuations of concentration $\sigma_{C}/\bar{C}$ increase with the distance from the plume centerline (Mylne and Mason, 1991; Yee et al., 1993; Justus et al., 2002) while the conditional fluctuations are approximately constant. This indicates that the changes along a horizontal cross-section of the plume are primarily caused by decreases in intermittency and, indeed, decreases in intermittency have been observed directly (Yee et al., 1993; Justus et al., 2002).

In theoretical modeling work, Celani and colleagues used a Lagrangian approach to solve the advection-diffusion equations and calculate intermittency as a function of downwind or crosswind distance^7^ from the source. They obtained a formula relating fluctuations to the intermittency factor, $x:\sigma_{C}/\bar{C}\approx \sqrt{x^{-1}-1}$ (Celani et al., 2014). When combined with results on the dependence of $\sigma_{C}/\bar{C}$ on downwind and crosswind distance, this predicts that intermittency decreases in the crosswind direction, in agreement with the experiments, but is independent of downwind distance. Celani et al. backed their results by showing that the value of $\sigma_{C}/\bar{C}$ is approximately constant, at long distances, for a subset of the experimental results (“Fens,” Figure 10 in Mylne and Mason, 1991, see Figure 4B). However, the fluctuations are not constant for other data sets at shorter distances (see “Sirhowy valley,” Figure 10 in Mylne and Mason, 1991, Figure 5 in Fackrell and Robins, 1982a, Figure 2 in Yee et al., 1993, and Table 1 in Yee et al., 1995) as it is evident from comparing their Figure 4A vs. Figure 4B (Celani et al., 2014; or see Figure 4A). Moreover, for the same dataset, intermittency was empirically shown to be around 0.3 for distance around 75 m (in qualitative agreement with Celani et al. prediction), but it increased substantially for distances around 750 m (see Figures 11, 13 in Mylne and Mason, 1991; Figure 4D). The causes of these discrepancies remain to be determined, in particular whether they are experimental in nature or rooted in the assumptions of the theoretical work.

In the windtunnel, for much smaller distances (>30 cm), and turbulent conditions (wind speed 10 cm/s and turbulence grid in the upwind end) the intermittency factor is strongly dependent on the downwind distance (see Figure 8 of Connor et al., 2018) both for high and low turbulence conditions.

##### Whiff and blank durations

When looking at whiff and blank durations individually, we observe an interesting U-shaped behavior of the average duration of whiffs and upcrossing times (duration of a whiff and the subsequent blank) with downwind distance with a minimum value at around 60 m (Yee et al., 1995; Figures 5, 6). This likely reflects that close to the source, the filaments^8^ of the plume are not yet broken up as much by turbulence and hence whiffs are long. At the minimum, filaments are fully broken up by turbulence, leading to minimal whiff duration. At even larger distances, the ongoing spreading of filaments begins to dominate, which increases the whiff duration again. Interestingly, this result is independent of the concentration threshold used to define whiffs/filaments, for a reasonably wide range of concentrations (Yee et al., 1995).

For distances between 60 and 330 m we also have information about the full distribution of whiff and blank durations. Theory and experiments indicate that, in this range and for whiff/blank durations between 10^–2^ and 10^2^ s, the probability distributions are independent from the concentration threshold and the downwind distance, following a power law with exponent -3/2 (Yee et al., 1995; Celani et al., 2014). The differences in the mean values for longer distance discussed above stem from additional very rare but very long whiffs/blanks while the distribution of short whiffs remains essentially the same.

Remarkably, the maximum of the probability distribution for the whiff duration is at very short timescales, around 3 ms and remains like this for long distances (at least between 60 and 330 m; Yee et al., 1995, see Figures 4E,F). This indicates that even though filaments “bleed out” at longer distances, there is always a strong element of very fast fluctuations.

Unfortunately, for time intervals below 10^–1^ s, the experimental evidence is hard to interpret because: (1) While the apparatus of Yee et al. had a sample rate of up to 270 Hz, it also had a loss of 6 dB in sensitivity at the smallest measurable timescale (~3 ms). (2) The minimal length scale of fluctuations – described by the Batchelor scale (see Box 2) and calculated from the value of kinematic viscosity of air and the energy dissipation rate furnished by Yee et al. (1995, Table 2) – is approximately 0.3 mm, which, for wind speeds around 1 m/s implies possible fluctuations on timescales as low as 0.3 ms (Yee et al., 1995).

Up to 1995, there was neither a mathematical model for the probability distribution of whiffs and blanks, nor for the amplitude of concentration (today there is the above mentioned work of Celani et al., 2014). Therefore, Yee et al. (1995) decided to simply fit the experimental distributions with a number of standard two-parameters probability distribution functions (e.g., the lognormal distribution, the gamma distribution, the conjugate beta distribution, the K-distribution, the Weibull distribution, and the Gumbel distribution). From a qualitative analysis of quantile-quantile plots, the authors observed that the best fit for the whiff durations was achieved with a lognormal distribution. This implies that the processes behind filament durations and arrival times are not memoryless^9^, for otherwise their distribution should follow an exponential distribution (see e.g., Gallager, 1996). The relationship between duration/arrival time and the amplitude of the whiffs is still unknown.

Recently, it has been shown that the frequency of bouts (significant changes in the odorant signal) can be used to determine, in a wind tunnel, the distance of the detector from the source (Schmuker et al., 2016).

##### Environmental features shaping plume structure

Aside from the down- and crosswind distance discussed so far, other factors such as wind speed, source position, source size and environmental conditions also have been investigated.

As explained above, *wind speed* is directly related to the degree of air turbulence as reflected by the Reynolds number (Re and the average wind speed are linearly related). Empirical evidence showed that faster wind (higher turbulence) yields thinner plume filaments (Yee et al., 1993) and a higher frequency of whiffs (Fackrell and Robins, 1982a).

The *source position* influences the temporal characteristics of plumes: A source located in a higher position will be affected by stronger advective flows than sources located closer to the ground, where “no-slip” boundary conditions constrain the flow to zero advection (see Connor et al., 2018; and Box 2).

The *source size* also has a significant influence on the plume structure. For small distances (within wind tunnel spatial scale, i.e., a few meters), experimental and theoretical results showed that for increasing source size, fluctuations decrease and intermittency increases [e.g., Figures 3, 4 from Fackrell and Robins, 1982b and Equation (9) of Celani et al., 2014]; theoretical analysis from Riffell et al. (2008) predicts that the source size affects the frequency of the eddies emitted. In principle, source size could even influence the plume statistics at long distances, but theoretical work of Celani et al. (2014) predicts that it does not affect any macroscopic measurements (average concentration in a whiff, intermittency, distribution of whiff duration, etc.).

*Environmental conditions* (via buoyancy), as mentioned before, affect the plume structure, in terms of average concentration, intensity and intermittency, but the experimental results are mixed. In Mylne (1992), the authors showed no difference for the intensity between the stable and near-neutral buoyancy case, while (Mole and Jones, 1994) showed that intensity is higher for stable than for unstable conditions: Stable conditions lead to higher average concentration and standard deviation than unstable conditions, but when normalized to the wind speed the differences are not significant (Mole and Jones, 1994).

##### Habitat

Contrary to flat environments like meadows or deserts, forests and other more structured environments are spatially complex and the boundary layer assumptions are not valid for them (see e.g., Aylor et al., 1976; Riffell et al., 2008); for example, large eddies are not present due to the canopy and the tree trunks, while vertical variations of the habitat are more relevant (Rauner, 1976; Hutchison and Baldocchi, 1989). Air movements due to advection are very small and therefore odor plumes are trapped into the canopy (Thistle et al., 2004). Of course, in this habitat, odorant propagation is much more difficult and even before reaching 100 m distance to the source, concentration values are typically already below 0.1% of the original values (Thistle et al., 2004). Moreover, the odor background in these environments generates even more difficulties to detect an odor of interest and how insects can cope with them is an active research area (Gorur-Shandilya et al., 2017; Erskine, 2018; Sehdev and Szyszka, 2019).

#### Mixtures of Odorants

“Experimental science hardly ever affords us more than approximations to the truth; and whenever many agents are concerned we are in great danger of being mistaken.”

**H. Davy, 1778–1829**

Mixtures of odorants have at least two levels of complexity that together generate the “olfactory cocktail party” problem (Rokni et al., 2014):

1. Odor responses are generally broad and overlapping: Individual chemical compounds with a defined meaning are rare exceptions and for them, early sensory areas work through dedicated paths called “labeled lines.” For instance, in *Drosophila* there is a single dedicated glomerulus for CO_2_ (Suh et al., 2004) (but see the recent results in van Breugel et al., 2018, and one for geosmin; Stensmyr et al., 2012). Apart from these exceptions, each odorant activates a broad profile of olfactory receptor types and each receptor type is activated by a broad profile of odorants.
2. Natural odors are mixtures of many odorants: plants and animals do generally not exude single odorants (with the exception of some pheromones) but multiple odorants at the same time. For example, floral scents can comprise more than 100 relevant odorants (Riffell, 2012; Beyaert and Hilker, 2014). It is the joint effect of these odorants that elicits the behavioral response and there is a large amount of evidence that the information about the identity or the state of the source is contained in the ratio of the odorants in the mixture (see e.g., Visser and Avé, 1978; Christensen et al., 1989; Dorn et al., 2003; Bruce et al., 2005; Baker, 2008; Najar-Rodriguez et al., 2010, and the references therein).

Mixture processing has been the subject of numerous studies in ants, bees, flies and many other insect models and while an extensive review of mixture processing in insects would go beyond the scope of this review, the major issues analyzed in the last 20 years in this field are:

1. *Olfactory coding (*Galizia et al., 1999; Carlsson et al., 2002; Dobritsa et al., 2003; Guerrieri et al., 2005; Wright et al., 2005; Deisig et al., 2006; Ito et al., 2009; Olsen et al., 2010; Andersson, 2012; Lei et al., 2013; Martin et al., 2013),
2. Difference between food related receptors and pheromone receptors (van der Goes van Naters and Carlson, 2007; Wee et al., 2016),
3. *Odorant valence* (e.g., Voskamp et al., 1999; Riffell et al., 2009a; Leonard et al., 2011; Najar-Rodriguez et al., 2011; Andersson, 2012; Thoma et al., 2014; Badel et al., 2016; van Breugel et al., 2018; de Vreese and Martinez-Ortiz, 2018; Mohamed et al., 2019),
4. *The representation of the time course* (Broome et al., 2006; Su et al., 2011; Stierle et al., 2013; Martelli and Fiala, 2019),
5. *The comparative analysis between species* (Andersson et al., 2011; Clifford and Riffell, 2013),
6. *Complex overlapping plumes* (Broome et al., 2006; Myrick et al., 2009; Su et al., 2012),
7. *Learning* (Perez et al., 2015; Schubert et al., 2015),
8. *Specific effects, for example the non-synaptic interaction between ORNs* (Su et al., 2012; Zhang et al., 2019).

Here, we will focus on odor source separation and in this section, we will review experimental and theoretical results for the two most elementary situations: when two odorants are emitted from two separate sources and when they are emitted from the same, single source. Of course, this is just one of the possible starting points before approaching more complex situations with multiple odorants and multiple sources (see for example, on this same issue; Conchou et al., 2019). It is important to note that the technical difficulty of measuring two odorants simultaneously and in the same location is still a big obstacle to making further progress in this field. We will see below that several clever strategies have been developed to overcome this difficulty, for example adopting the insects’ antennae to detect the odorants (Loudon, 2003; Myrick and Baker, 2011).

##### Two sources, two odorants

When two odorants are emitted from two sources, they start off separated, but after a while and downwind from the sources, they mix due to diffusion and turbulent motion. In mathematical terms, the correlation of the time courses of the odorants’ concentrations increases with the downwind distance from the sources. Increasing the distance between the sources, this correlation decreases. Therefore, close to the sources, the odorants can be perceived as having been emitted from separated sources, but far downwind from the sources they cannot. If the distance between sources is higher, it is easier to discriminate whether they are separated or not.

A recent theoretical study (Kree et al., 2013) demonstrated that the correlation between concentrations emitted from two sources decreases exponentially for increasing inter-source distance and increases exponentially with the distance to the sources.

Davies et al. presented the first evidence of this phenomenon for large distances (source separation around 0–40 m, downwind distance 5–20 m, in near-neutral conditions, wind speed around 2 m/s). Interestingly, they adopted and modified two different detectors to obtain co-localized synchronous odorant measurements (Davies et al., 2000).

The recent work of the group of Schäfer analyzed the effect in a windtunnel on a smaller spatial scale (source separation around 0–50 cm, downwind distance 40 cm, air speed around 552 cf/m^10^). With Aurora Scientific they developed the first “dual-energy photoionization detector” and recorded the evolution of odorant concentration emitted from two sources, either mixed together or separated (Erskine, 2018; Erskine et al., 2019). The analysis of temporal correlation of the odor signal showed that “source separation” can be accurately predicted. Similar results were obtained with an odorant detector formed from four moth antennae (Myrick et al., 2009). In a wind tunnel of 1.5 m length, the detector was able to discriminate between plumes emitted from a single source from those coming from two closely spaced sources (2–10 cm separation). These are encouraging results that bring our technology a step closer to the performance of insects’ olfactory system: 20 years ago, Baker et al. tested moths with a mixture of a binary pheromone blend and an interspecific compound (a pheromone antagonist; Fadamiro and Baker, 1997) and observed that they are able to discriminate between a single source emitting the mixture and two sources emitting the same odorants even when separated by only 1 mm (Baker et al., 1998). Interestingly, this experiment appears to have never been repeated.

##### One source, two odors

When two odorants are emitted from a single source, each with a given, constant concentration, the ratio of their concentrations is informative of the source identity (as noted above), but very far from the source, due to diffusion and turbulent motion, the two odorants are spread out, their concentration ratio changes and the information about the identity can get lost. The most pertinent question in this scenario is to what extent do odorants initially travel together in the same filaments maintaining the same ratio of concentrations? And if they do so, for how long? And are the mixing effects due to diffusion and advection in a turbulent flow synchronous or do they take place at different timescales and hence take effect at different distances from the source?

The answers to these questions will depend on the physical properties of the flow, on the chemical properties of the odorants and on the differences between them (for an excellent review on this issue see Conchou et al., 2019).

For example, compounds with lower adsorbing properties would travel over longer distances (and faster) than the other compounds (see Beyaert and Hilker, 2014, and references therein). This effect can have a potential function as the ratio of the two components can inform the insect of the distance from the source. For example, Xiao et al. (2012) showed that two long-chain of hydrocarbons help orientate the yellow peach moth Conogethes punctiferalis (Crambidae) to a source, but only at close range (less than 3 m).

It is generally believed that the diffusive properties of odorants are not relevant for this particular issue (Celani et al., 2014; Cardé, 2016) because for most relevant odorants (e.g., pheromones) the Peclét number is much bigger than one, so that advection dominates over diffusion and the diffusivity of common odorants is quite similar and spans a range of only one order of magnitude. For example, the diffusion coefficient for ethanol is around 10^–5^ m^2^/s and that for hexadecanol (as many moth pheromones) is around 10^–6^ m^2^/s (Loudon, 2003); within a pheromone blend, the difference in diffusion coefficients is even less (Cardé, 2016).

Some indirect evidence supporting this hypothesis is presented in Duplat et al. (2010) who compared temperature and concentration profiles in plumes released in a sustained turbulent medium at several distances downstream from the source. They considered temperature and odorant concentration interchangeably as they are obeying the same type of advection-diffusion equations. In particular, they showed how the profiles of the relevant scalars (temperature or odorant concentration) change for three conditions with very different values of the Schmidt number (the ratio between Péclet number and Reynolds number, see Box 2). They analyzed temperature in air Sc = 0.7, temperature in water at Sc = 7, and the concentration of disodium fluorescein in water at Sc = 2000. In spite of the large differences in Schmidt number, the differences in the profiles for these three cases (diffusivity spans four orders of magnitude) are quite subtle.

### Application to Insect Olfaction Research

#### Navigating Odor Plumes

“Information is where you find it”

Dusenbery, 1996

The goal of insects navigating an odor-landscape is to approach or escape the odorant source. To this aim, insects must be able to “read” the plume in which they are immersed. In a previous section, we saw how the statistical properties of plumes vary depending on the source position, sensor position, temperature, wind speed, etc. Which of these pieces of information about the plume structure could potentially help insects? And which ones do they actually use? Do insects analyze and extract information from the complex structure of odor plumes as recently suggested in Boie et al. (2018) or do they use only relatively simple cues, like the presence or absence of an odorant at any given time (Pang and Farrell, 2006)? In this final section, we would like to show the relevance of these questions for the study of insect navigation. To this aim, we will use only a few illustrative examples from the literature. For an extensive review of odor-guided insect navigation (see e.g., Murlis et al., 1992; Belanger and Arbas, 1998; Vickers, 2000; Moore and Crimaldi, 2004; Gaudry et al., 2012; Cardé, 2016; Webster and Cardé, 2017; Baker et al., 2018).

We saw that both downwind and crosswind distance from the source affect intermittency, average concentration, and frequency of bouts (see e.g., Mylne and Mason, 1991; Yee et al., 1993; Schmuker et al., 2016). However, we also saw that their isolated local values (of intermittency and average concentration) prevent to unambiguously determine the distance to the source. For example, for distances over 60 m the excursion times have very similar probability distributions (known up to 300 m), with relevant differences only for very long excursion times (>1 s, see Figures 4E,F). Therefore, to know the distance from their objective, insects must integrate information across space and/or time. In a recent experiment, Pang et al. (2018) demonstrated the effect of memory on olfactory guided orientation decisions of flies and mosquitoes in a laminar flow within a windtunnel. Another example is the dependence of turbulence in the atmospheric boundary layer on the time of day: during the sunset there are less advection movements, and as a consequence plumes intermingle less (Mylne and Mason, 1991; Yee et al., 1993; Mole and Jones, 1994). If insects wanted to use measures of turbulence for orientation, they would need to adjust this information for the time of day. It has even been suggested that this effect influenced, via evolutionary selection, the circadian rhythm of the moth *manduca sexta* that exhibits nocturnal foraging (Riffell et al., 2009b), presumably in order to take advantage of the more stable conditions during the night.

There are several strategies that motile organisms developed to locate an odorant source (Belanger and Arbas, 1998; Vickers, 2000; Moore and Crimaldi, 2004; Gaudry et al., 2012). A first classification of potential strategies can be performed based on the level of turbulence of the flow that the animals encounter.

For example, at low Péclet number and low Reynolds number, diffusion processes dominate the flow dynamics (see Figures 3A,B) and animals follow the gradient of odor concentration (chemotaxis). This strategy can range from simple biased random walks of bacteria (Weissburg, 2000) to more sophisticated active sampling behaviors observed in *Drosophila* larvae (Gomez-Marin et al., 2011). We saw that close to the surface, the no-slip boundary condition generates a layer of low speed (Connor et al., 2018) and it is well-established that *Drosophila* larvae use resulting odor gradients (Louis et al., 2008). It has also been suggested that walking insects could take advantage of the diffusive distribution of odorants (Baker et al., 2018). However, the odor landscape is patchy even for animals relatively close to the surface, like ants (few mm high), and already at small distances (>30 cm) from the source (Figure 8 in Connor et al., 2018). This is also reflected in the trajectories of desert ants which frequently change between upwind and crosswind directions, presumably because they constantly get into and out of the plume (Buehlmann et al., 2014, 2015).

For increasing advective wind, the odor-landscape becomes turbulent (high Reynolds number, high Péclet number) (see Figures 3C,D). In these conditions the patchiness of plumes prevents insects from using any gradient based information (Wright, 1958; Gifford, 1959; Aylor et al., 1976; Elkinton et al., 1984) and insects have to smell and navigate based on the pattern of discontinuous stimulation. Indeed, the behavioral relevance of stimulus intermittency has been repeatedly shown (Kennedy et al., 1981; Willis and Baker, 1984; Baker et al., 1985; Bjostad, 1987; Kaissling, 1997) together with empirical demonstrations of the correlation between AL intermittent responses and insect (in moth) navigation behavior (e.g., Lei et al., 2009; Huston et al., 2015). To react to plume intermittency, insects have to be fast: Indeed, for more than 20 years we have known that insects can extract information at small time scales (below 1 s; Mafra-Neto and Cardé, 1994; Vickers and Baker, 1994; Baker et al., 1998), and it is now becoming clear that even fluctuations of around 10 ms can be detected (Bhandawat et al., 2010; Szyszka et al., 2012, 2014). In a turbulent plume, insects go into and out of a plume frequently, so that in addition to locating the source, just (re-)locating the plume becomes challenging. Typically, their behavior can be described as alternating upwind surges when in a whiff and (approximately) crosswind casting during blanks (David et al., 1983; Kuenen and Cardé, 1994; Buehlmann et al., 2014; van Breugel et al., 2014). In the presence of wind, the plumes tend to be elongated along the wind direction. It can then appear intuitive to think of crosswind casting as a good approach to re-locating the plume once it has been lost. However, over the years different “optimal” models have been proposed to capture the dynamics of this behavior (Dusenbery, 1989). Unfortunately, we are still missing a complete representation of what different insects actually do when losing the plume.

#### Scaled-Down Odor Plumes in the Lab

Odor plumes are too complex to be used in their full details when investigating animal physiology and behavior and hence need to be simplified. Odor steps are the most radical simplification, are very easy to generate and analyze, and are, therefore, the most common inputs used in insect neurophysiology until today – with some exceptions starting 10 years ago (see e.g., Geffen et al., 2009). However, constant odor steps do not occur in natural plumes, whose most important characteristic is intermittency. In a recent study Jacob et al. (2017), recorded activity in the early olfactory sensory areas (olfactory receptors and antennal lobe) in a controlled environment using olfactory stimuli imitating the distribution of whiffs and blanks of natural plumes (a similar example is Huston et al., 2015), but simplifying the concentration to a constant value. The goals of this seminal work reflect the same thinking underlying this review: the complexity of olfactory stimuli has to be faced altogether because a reductionist approach might lead to misunderstanding the olfactory system. The introduction of correct whiff and blank statistics is a great first step and there are several further improvements that can be made: (1) Removing the approximation of a single value for the concentration, for the obvious reason that otherwise ORNs or antennal lobe neurons cannot exhibit realistic responses; (2) Implementing simulated stimulation for crosswind distances different from zero, (3) Measuring the plume-structure for very small time-scales; as discussed earlier, the distributions are not known for the time-scales below tens of milliseconds. (4) Measuring the whiff and blank distributions perceived by a moving insect: Whiff and blank distributions are extracted from a stationary point, subject to the passing of the plume, but insects usually navigate actively into plumes with a speed that is comparable with the wind speed. It is reasonable to expect different distributions of whiffs and blanks for this situation. To solve the first and second issues, no further data are needed, one can implement the results from existing experimental studies (Mylne and Mason, 1991; Yee et al., 1995) but for the other two issues, further experiments are needed.

## Conclusion and Future Directions

“To advance further, we must continue our trend of placing the animal firmly in its fluid mechanical environment and probing more finely the properties of fluid flow and signal structure that have significant impacts on locomotory performance”.

**M. J. Weissburg, Biol. Bull. 2000**

“[…] while the insect’s powers of olfaction are remarkable they are not miraculous”.

**H. Wright, 1958**

We have summarized some knowledge on the nature of odor stimuli touching on two specific aspects: (1) The concentration of odorants emanating from liquid dilutions and the temporal structure of odor stimuli produced by olfactory stimulators in the lab. (2) The structure of odor plumes in natural environments.

With respect to the odor concentration produced by using defined dilutions of odorants in a solvent, it is important to reiterate that the concentration in the air within the headspace of the liquid solution, be it on a filter paper or in a larger reservoir, is not a linear function of the dilution. As we have discussed and as is well-known there are linear regimes for the two extremes of very high dilution (very small amounts of odorants, Henry’s law) and for very low dilution (almost pure odorant, Raoult’s law). What may be less appreciated are the regimes where these laws apply. In a sense, both regimes are very small if looked at on a linear scale (see e.g., Figure 2): Henry’s law typically applies from dilutions of 10^–2^ onward, while Raoult’s law might apply for 90% or more odorant in the solution. However, given the typical goal of olfaction research to investigate realistic, diluted concentrations all the way down to the detection threshold, Henry’s law typically applies and future research should make appropriate use of Henry’s constant, not the vapor pressure. In order to do so, we need to extend the data on Henry’s constant which is currently only available for a few odorant-solvent pairs. Independently, it will always remain important to ascertain using direct measurements, e.g., with a PID device, that the stimuli we think we have generated by diluting odorant solutions, are indeed what we expect them to be.

With respect to the temporal structure of odorant stimuli from odor stimulation devices, we discussed recent results showing that the odor onset of an odor stimulus depends on the identity of the odorant. Combined with other results that indicate that olfactory systems are sensitive to the derivative of the odorant concentration as well as the odorant concentration itself, the difference in odor onset slope could have measurable effects on the response and this could lead to confusing results. As with the odorant concentration, it should also become standard to ascertain the stimulus time course for any given experiment. Research in insect physiology is clearly moving toward more articulate stimuli – more odorants, more complex time courses. Moreover, nowadays it is clear that a purely reductionist approach is insufficient to gain a full understanding of the neuroscience of insects – from molecules, to neurons and synapses, and to behavior. Future experiments may eventually all have to consider the entire environment-perception-action loop (Wallach et al., 2016), including, for instance, how wing movements might implement strategies for active sensing of odor plumes (Koehl, 2006; Li et al., 2018). However, it is an immediate objective for the community to define protocols for more viable and precise spatio-temporal stimulus generation in the lab (e.g., Gorur-Shandilya et al., 2019).

For the structure of natural odor plumes we surprisingly found that many of the most salient experimental results date back to the last century, only augmented by occasional more recent studies. Even in the quite simplified overview that we were able to include here it becomes clear that ultimately we still do not fully understand the nature of odor plumes. A prime example is the measurement of intermittency, probably one of the most important plume descriptors, where theoretical results are in stark disagreement with the experimental evidence. Solving this issue is a well defined goal that should urgently be addressed by Neuroscientists and Physicists alike.

There is increasing evidence and acceptance that relevant temporal and spatial scales can be quite small, matching the recent discoveries of “fast olfaction” (see section Application to Insect Olfaction Research). When investigating insect behavior in natural plumes, in particular navigation, it will be important to better understand and experimentally characterize the particular plume conditions that insects face in any particular experiment. To this aim, a big technological effort is needed to measure odor mixtures at a high spatio-temporal resolution (Davies et al., 2000; Myrick et al., 2009). This could then feed into lab experiments for which advanced stimulation devices for arbitrary time series are under development (Kim et al., 2011, 2015; Jacob et al., 2017; Gorur-Shandilya et al., 2019).

Generally we desperately need more data and it will be essential to update the decades old data on natural odor plumes in order to make further progress in our understanding of insects’ behaviors in natural environments.

## Author Contributions

MP and TN contributed to research and analysis of literature and writing the manuscript.

## Conflict of Interest

The authors declare that the research was conducted in the absence of any commercial or financial relationships that could be construed as a potential conflict of interest.

## Footnotes

1 The debate about the dimensionality of olfaction: Bushdid et al., 2014; Gerkin and Castro, 2015; Meister, 2015).

2 The proposal of separating olfaction in multiple senses is not so absurd: magnetoception is distinguished from vision, and hygroreception is already separated from olfaction.

3 If we are interested in the receptor response, the variable of interest if the concentration, but of course, if we consider the system as a whole – odor-source plus animal – the variable of interest could also be the dilution.

4 See for example https://pubchem.ncbi.nlm.nih.gov or http://www.chemspider.com/.

5 Note that some materials also adsorb odorants on very long time scales and lead to long term contamination, which can be an issue in olfaction research but we will not consider it here.

6 Sometimes also referred to as “fluctuation intensity” or “relative fluctuations of concentration.”

7 The distance from the plume centerline (which points along the wind direction).

8 A filament is understood in this context as a contiguous area with non-zero odorant concentration.

9 The exponential distribution uniquely has the property of being memoryless: if the upcrossing times were exponentially distributed, a fly that met a whiff 1 s ago, would have the same expectation to find another whiff in the near future as a fly that met a whiff 1 ms ago.

10 CFM is short for cubic feet per minute (cu ft/min). It is a measurement of the velocity at which air flows into or out of a space.
